# Keep Your Feet Warm

**Published:** 1875-12

**Authors:** 


### KEEP YOUR FEET WARM.

To keep these extremities warm is to effect an insurance against the almost interminable list of disorders which spring out of a "slight cold." First, never be tightly shod. Boots or shoes, when they fit closely, press against the foot, and prevent a free circulation of blood. When, on the contrary, they do not embrace the foot too tightly the blood gets fair play, and the spaces left between the leather and the stocking are filled with a comfortable supply of warm air. The second rule is, never sit in damp shoes. It is often imagined that unless they are positively wet it is not necessary to change them while the feet are at rest. This is a fallacy; for when the least dampness is absorbed into the sole it is attracted further, to the foot itself, by its own heat, and thus perspiration is dangerously checked. Any person may prove this by trying the experiment of neglecting the rule, and his feet will become cold and damp after a few moments, but on taking off the shoe and warming it, it will appear quite dry.

DIPHTHERIA.— From many years' experience in the treatment of diphtheria, writes a physician of Boston, I am led to believe that the most useful counter-agent that can be employed is the inhalation of the vapor of carbolic acid. In my earlier practice I employed nitrate of silver as a topical application by brushes and laryngial syringes, which I found useful as others have done; but the difficulty of application in some cases was a serious limitation to its usefulness; therefore I adopted the use of carbolic acid in the following manner: A piece of cloth is wet with the acid, and being loosely thrust into a small lamp chimney or other similar tube, the air is drawn through this and the air passages to the air cells of the lungs, where an antiseptic influence seems to be exerted upon the blood. Many cases that had resisted other treatment yielded readily to this, and the nitrate of silver was wholly laid aside. The only other special treatment required is the frequent use of small portions of chloride of iron, alternated with quinine, while the general system is sustained by a generous diet of beef tea, whole-wheat bread, etc.

To those who enjoy a blazing wood fire, and with its shining brass andirons, we reeommend the following method of polishing until the gilded ball on every one will present many phases:—"First use a cotton cloth saturated with vinegar to rub off the tarnished stains, and then wipe dry; after this is done take a flannel cloth and polish them with dry whiting; and the result will be a white glittering appearance, calling forth the admiration of every one who enters the apartment they adorn.

